# Study on the pharmacokinetics, tissue distribution and excretion of laurolitsine from *Litsea glutinosa* in Sprague-Dawley rats

**DOI:** 10.1080/13880209.2021.1944221

**Published:** 2021-07-03

**Authors:** Yin-Feng Tan, Rui-Qi Wang, Wen-Ting Wang, Ying Wu, Ning Ma, Wei-Ying Lu, Yong Zhang, Xiao-Po Zhang

**Affiliations:** a Key Laboratory of Tropical Translational Medicine of Ministry of Education, Hainan Key Laboratory for Research and Development of Tropical Herbs, School of Pharmacy, Hainan Medical University, Haikou, P. R. China; b Reproductive Medical Center, Hainan Women and Children’s Medical Center, Haikou, China; c Department of Pharmacology, Hainan Medical University, Haikou, Hainan, China

**Keywords:** Aporphine alkaloid, LC-MS/MS

## Abstract

**Context:**

Laurolitsine is an aporphine alkaloid and exhibits potent antihyperglycemic and antihyperlipidemic effects in ob/ob mice.

**Objective:**

To investigate the pharmacokinetics, tissue distribution and excretion of laurolitsine.

**Materials and methods:**

A LC-MS/MS method was established and validated to determine laurolitsine concentrations in the biological matrix of rats (plasma, tissue homogenate, urine and faeces). 10 Sprague-Dawley (SD) rats were used for plasma exposure study: 5 rats were injected with 2.0 mg/kg of laurolitsine via the tail vein, and the other 5 rats were administered laurolitsine (10.0 mg/kg) by gavage. 25 SD rats used for tissue distribution study and 5 SD rats for urine and faeces excretion study: rats administered laurolitsine (10.0 mg/kg) by gavage. After administered, serial blood, tissue, urine and faeces were collected. Analytical quantification was performed by a previous LC-MS/MS method. The pharmacokinetics, bioavailability, tissue distribution and excretion of laurolitsine were described.

**Results:**

The pharmacokinetic parameters of oral and intravenous administration with *T_max_* were 0.47 and 0.083 h, *t_1/2_* were 3.73 and 1.67 h, respectively. Oral bioavailability was as low as 18.17%. Laurolitsine was found at a high concentration in the gastrointestinal tract, liver, lungs and kidneys (26 015.33, 905.12, 442.32 and 214.99 ng/g at 0.5 h, respectively) and low excretion to parent laurolitsine in urine and faeces (0.03 and 1.20% in 36 h, respectively).

**Conclusions:**

This study established a simple, rapid and accurate LC-MS/MS method to determine laurolitsine in different rat samples and successful application in a pharmacokinetic study.

## Introduction

Aporphine alkaloids are a type of alkaloids widely present in plants. There are more than 500 kinds of aporphine alkaloids that have been isolated and identified, many of which have anticancer (Lu et al. 2012; Ge and Wang 2018), antivirus (Montanha et al. 1995), anti-inflammatory (Marahel and Umesha 2016), and hypoglycaemic activity. Pharmacokinetics and oral bioavailability are important to select drug candidates to undergo clinical testing, in addition to providing a basis for the definition of an effective dosing regimen associated with adequate plasma concentrations (Kola and Landis 2004). Approximately 10 aporphine alkaloids have been studied for pharmacokinetics in animals, such as boldine (Cermanova et al. 2016), roemerine (Liu et al. 2014), cepharanthine (Deng et al. 2017), and isoboldine (Li et al. 2015). Laurolitsine is an aporphine alkaloid that has been isolated from many plants, such as *Illigera aromatica* S.Z. Huang & S.L. Mo (Hernandiaceae) (Ge et al. 2018), *Phoebe tavoyana* (Meissn.) Hook f. (Lauraceae) (Omar et al. 2020), and *Peumus boldus* Mol. (Monimiaceae) (Fuentes-Barros et al. 2018). Previously, we isolated an alkaloid-rich extract containing laurolitsine from *Litsea glutinosa* (Lour.) C. B. Rob. (Lauraceae) and found that the alkaloid-rich extract exhibited potent antihyperglycemic and antihyperlipidemic effects in ob/ob mice (Zhang et al. 2018). A single alkaloid of laurolitsine was obtained from *Litsea glutinosa* in our further research, and laurolitsine stimulated HepG2 liver cell glucose consumption *in vitro* and was not cytotoxic. At the same time, we also demonstrated that laurolitsine exhibited obvious hypoglycaemic and hypolipidemic effects in diabetic db/db mice *in vivo*. Laurolitsine could be a good candidate antidiabetic drug; its unique structure has attracted our full interest. Pharmacokinetic parameters are necessary for understanding biological effects and preclinical studies *in vivo*. However, the pharmacokinetics of laurolitsine were absent. In this study, an LC-MS/MS quantitative analysis method of laurolitsine was established, and the pharmacokinetics, tissue distribution and excretion through bile, urine and faeces in Sprague-Dawley (SD) rats after intragastric and intravenous administration of laurolitsine were determined. In brief, we aimed to calculate the relevant pharmacokinetic parameters of laurolitsine in rats, explain its pharmacokinetic characteristics *in vivo*, and lay a theoretical foundation for its further research and development.

## Materials and methods

### Material

Laurolitsine was separated and purified from the bark of *L. glutinosa* by our research group (Zhang et al. 2018). It was identified by NMR, MS and other spectral analyses, and the purity determined by HPLC was >98%. It was stored at −20 °C. Nuciferine was used as an internal standard and was provided by the National Institutes for Food and Drug Control. Methanol, acetonitrile, and formic acid were all chromatographically pure and provided by Aladdin Industrial Corporation. Purified water was prepared by a LabTower EDI system (Thermo Scientific, USA).

### Instruments and conditions

Triple Quad5500 triple quadrupole tandem mass spectrometer (AB SCIEX, Singapore), LC-20ADXR two-pump liquid chromatography (Shimadzu, Japan), Sartorius ME2355 electronic analytical balance; BRAND micropipette.

Chromatographic separation was achieved on a Synergi Fusion-RP 80 Å C18 (2 × 50 mm, 4 μm) chromatographic column (Phenomenex, USA) at 40 °C. The mobile phases consisted of solvent A (water containing 0.5‰ formic acid) and solvent B (acetonitrile containing 0.5‰ formic acid) with a gradient elution: 5% B (0 min), 5–35% B (0–3 min), 35–95% B (3–3.5 min), 95% B (3.5–5 min), 5% B (5.1–6.5 min). The flow rate was 0.3 mL/min, and the injection volume was 3 μL. The temperature of the autosampler was maintained at room temperature.

The mass spectrometer was operated in positive electrospray ionization (ESI) mode with selected multiple reaction monitoring (MRM) mode for all the analytes. The precursor-to-product ion pairs used for laurolitsine and IS nuciferine were *m/z* 314.2→265.1 (DP 80, CE 18) and *m/z* 296.3→265.2 (DP 80, CE 20). The optimized MS parameters were set as follows: collision gas (CAD) at 10 psi, curtain gas (CUR) at 45 psi, nebulizer gas (GS1) at 55 psi, heated by N_2_ gas (GS2) at 60 psi, ion spray voltage at 5500 V and temperature at 550 °C, and scan time 40 ms.

### Preparation of standard and quality control samples

Laurolitsine stock solution (10 mL) was prepared with a concentration of 1 mg/mL in methanol and store in a refrigerator at 4 °C for later use. An appropriate amount of laurolitsine stock solution was diluted step by step with methanol to obtain laurolitsine standard curve working solutions with concentrations of 10, 100, 1000, 5000, 10,000, and 20,000 ng/mL and laurolitsine quality control samples with concentrations of 30, 1200, and 15,000 ng/mL.

Laurolitsine standard (5 μL) curve working solution and quality control sample working solution (5 μL) were added, and 45 μL of rat blank plasma was added to configure the plasma standard curve sample with final concentrations of 1, 10, 100, 500, 1000, 2000 ng/mL and quality control samples of 3, 120, 1500 ng/mL. The urine standard curve samples and quality control samples were prepared in the same ways. The rat tissues were weighed and cut into pieces, added to 3 times the mass of normal saline, homogenized under ice bath conditions, and prepared corresponding standard curve samples and quality control samples with tissue homogenate according to the above method. After weighing the rat faecal samples, 4 times the mass of deionized water was added, the samples were homogenized under ice bath conditions, and the corresponding standard curve samples and quality control samples were prepared with faecal homogenate according to the above method.

An appropriate amount of a standard substance, nuciferine, was accurately weighed, diluted with methanol to prepare a 1 mg/mL internal standard stock solution, and stored in a refrigerator at 4 °C for later use. The internal standard stock solution was diluted with methanol to a 50 ng/mL solution just before use.

### Sample preparation

Plasma and urine can be directly sampled and processed. The tissue sample was weighed and cut into pieces, added to 3 times the mass of normal saline, and homogenized under ice bath conditions. After the faecal samples were weighted, 4 times their mass of deionized water was added and homogenized in an ice bath. Tissue and faecal homogenates were collected for further processing. A 50 μL biological matrix sample was precisely aspirated and combined with 10 μL of internal standard solution (nuciferine/methanol solution, 50 ng/mL); the sample was vortexed for 2 min to mix, then combined with 190 μL of methanol to precipitate the protein. After being vortexed for 2 min to make the precipitation complete, it was centrifuged at 13,000 rpm and 4 °C for 10 min in a low-temperature centrifuge; 150 μL of the supernatant was placed in a nitrogen blower to dry at room temperature. A methanol-water solution (50:50, *V/V*) was used to reconstitute the dried sample; it was then vortexed for 2 min and centrifuged at 13000 rpm for 10 min, and the supernatant was taken for analysis.

### Method validation

The analytical method was validated according to the bioanalytical method guidelines suggested by Chinese Pharmacopoeia (Chinese Pharmacopoeia Commission 2020). The specificity, linearity, sensitivity, carryover effect, accuracy, precision, matrix effect, recovery, dilution integrity and stability of the LC-MS/MS method established in rat plasma, liver, kidney and other tissues, urine, faeces and other biological matrices were verified.

Taking plasma samples as an example, the specific method verification is as follows. Comparing the LC-MS/MS spectra of blank plasma samples from 6 different rats, standard curve plasma samples and rat plasma samples after *i.v.* and laurolitsine to verify the specificity of the method. The carryover effect was evaluated by the analysis of blank samples following the ULOQ samples. Standard calibration curves were constructed by plotting the measured pear area ratios of laurolitsine to IS versus the concentration of laurolitsine. The lower limit of quantification (LLOQ) is considered the lowest calibration standard and can be quantified reliably, with acceptable accuracy (80–120%) and precision (<20%). The intra- and inter-day precision and accuracy were assessed by calculating the QC samples (3, 120, 1500 ng/mL) in six replicates on one day and on three days, respectively.

The post-extraction addition method was used to evaluate the matrix effect, and the extraction recovery rate was also investigated. According to the references (Chen et al. 2014), three different solvents were used to prepare laurolitsine samples with high, medium and low concentrations (3, 120, 1500 ng/mL), and 6 parallel samples were used for each concentration. *Set 1* series sample solvent is methanol; the *Set 2* series sample solvents are blank plasma from different rats, supernatants obtained by centrifugation after 4 times the volume of methanol to precipitate proteins; *Set 3* series sample solvents are derived from different rats with blank plasma. Fifty microliters of each of the above samples was treated according to the method under "Sample preparation", and the samples were analyzed according to the method under "Instruments and conditions". The peak areas are *A_1_, A_2_*, and *A_3_*, and the matrix factor MF= *A_2_/A_1_*×100%; extraction recovery rate RE= *A_3_/A_2_*×100%. The normalized matrix factor is the ratio of the matrix factor of laurolitsine to IS.

The stability of the high-, medium-, and low-concentration QC samples under four conditions was investigated: placed in a refrigerator at 4 °C for 24 h, after three repeated freezing-thawing cycles (12 h/12 h), placed in a refrigerator at −20 °C for 7 days, and placed at room temperature for 6 h.

### Plasma exposure study

Male SD rats (200 ± 20 g) were purchased from Hunan Slack Jingda Experimental Animal Co., Ltd. (animal production licence number: SCXK (Xiang) 2018-0012). Ten healthy SD rats were randomly divided into two groups, five in each group. After 12 h of fasting, the rats in the two groups were given laurolitsine 10 mg/kg by gavage, and laurolitsine 2 mg/kg was injected into the tail vein. SD rats were anaesthetized with isoflurane, and blood samples were collected from the orbital vein at 0, 0.083, 0.167, 0.333, 0.667, 1, 1.5, 2, 4, 6, 8, and 10 h after laurolitsine administration. The blood sample was placed in a centrifuge tube pre-treated with sodium heparin and centrifuged at 4000 rpm for 10 min, and the supernatant was collected and stored in a refrigerator at −20 °C. The plasma samples were processed according to the method under "Sample preparation" and analyzed according to the method under "Instruments and conditions" to determine the concentration of laurolitsine in rat plasma at different time points. DAS 3.2.8 pharmacokinetic software was used to process data, calculate relevant pharmacokinetic parameters and draw a blood drug concentration-time curve.

### Tissue distribution study

After fasting for 12 h, 25 SD rats were randomly divided into 5 groups, each with 5 rats. The rats received 10 mg/kg laurolitsine through oral administration. Tissue samples, including heart, liver, spleen, lung, kidney, stomach, small intestine, brain, muscle, fat, and testis, were collected 0.5, 1, 2, 4, and 6 h after dosing in the five groups. All samples were stored at −80 °C after weighing. Process and analyze according to the aforementioned method, and determine the concentration of laurolitsine in each tissue sample at different time points.

### Urine and faeces excretion studies

Five male SD rats were placed in 5 metabolic cages for one day. Blank samples of urine and faeces were collected. Laurolitsine 10 mg/kg was given by gavage to each rat. After administration, urine and faeces samples were collected from the rats at 0–4, 4–8, 8–12, 12–24, and 24–36 h. The samples were processed and analyzed according to the aforementioned methods to determine the laurolitsine content in urine and faecal samples at different time periods.

## Results

### Method validation

#### Specificity and carryover

**Figure 1.**
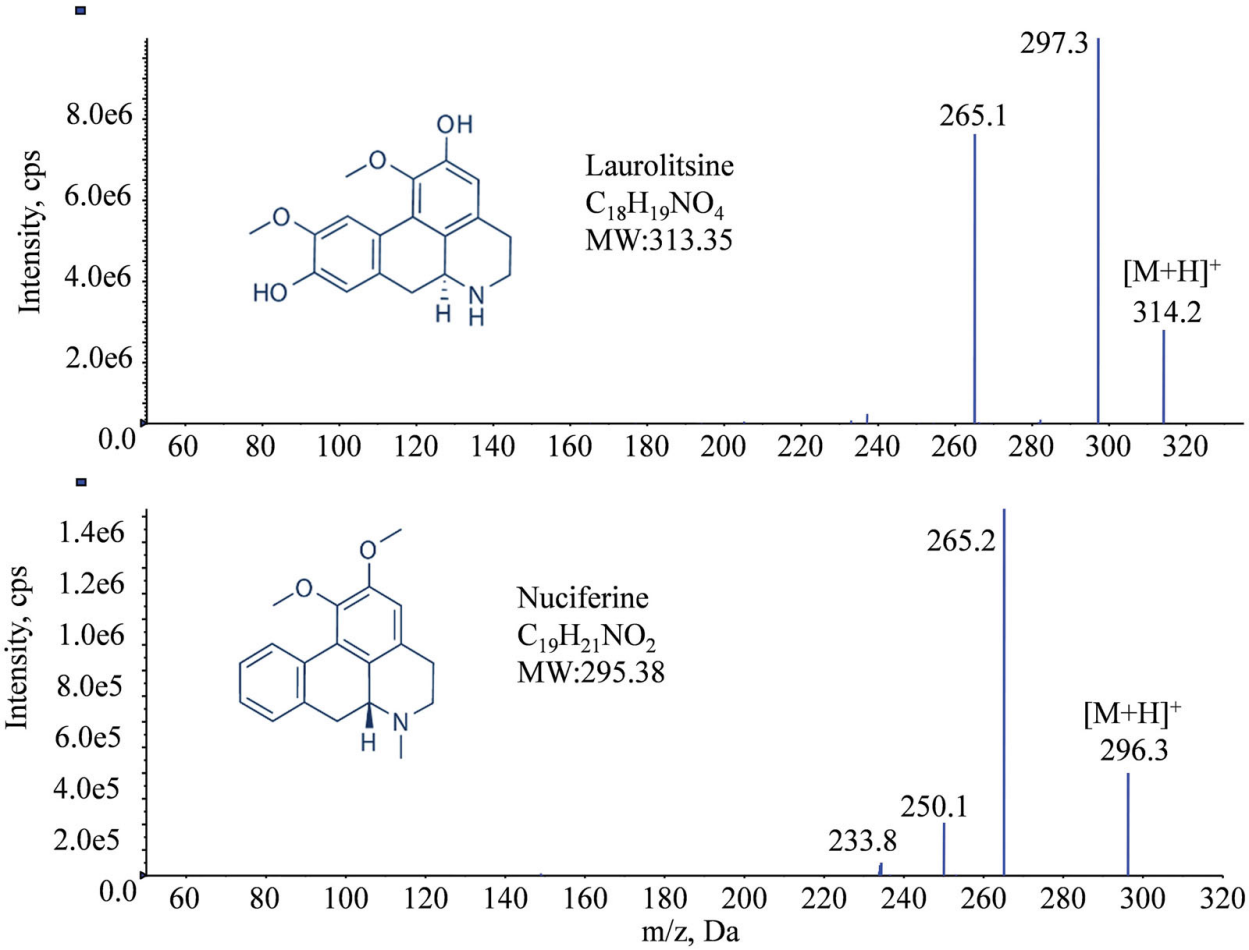
Chemical structures and MS/MS spectra of laurolitsine and nuciferine.

The MS/MS spectra of laurolitsine and nuciferine are shown in Figure 1 and were consistent with the theoretical molecular weight.

**Figure 2.**
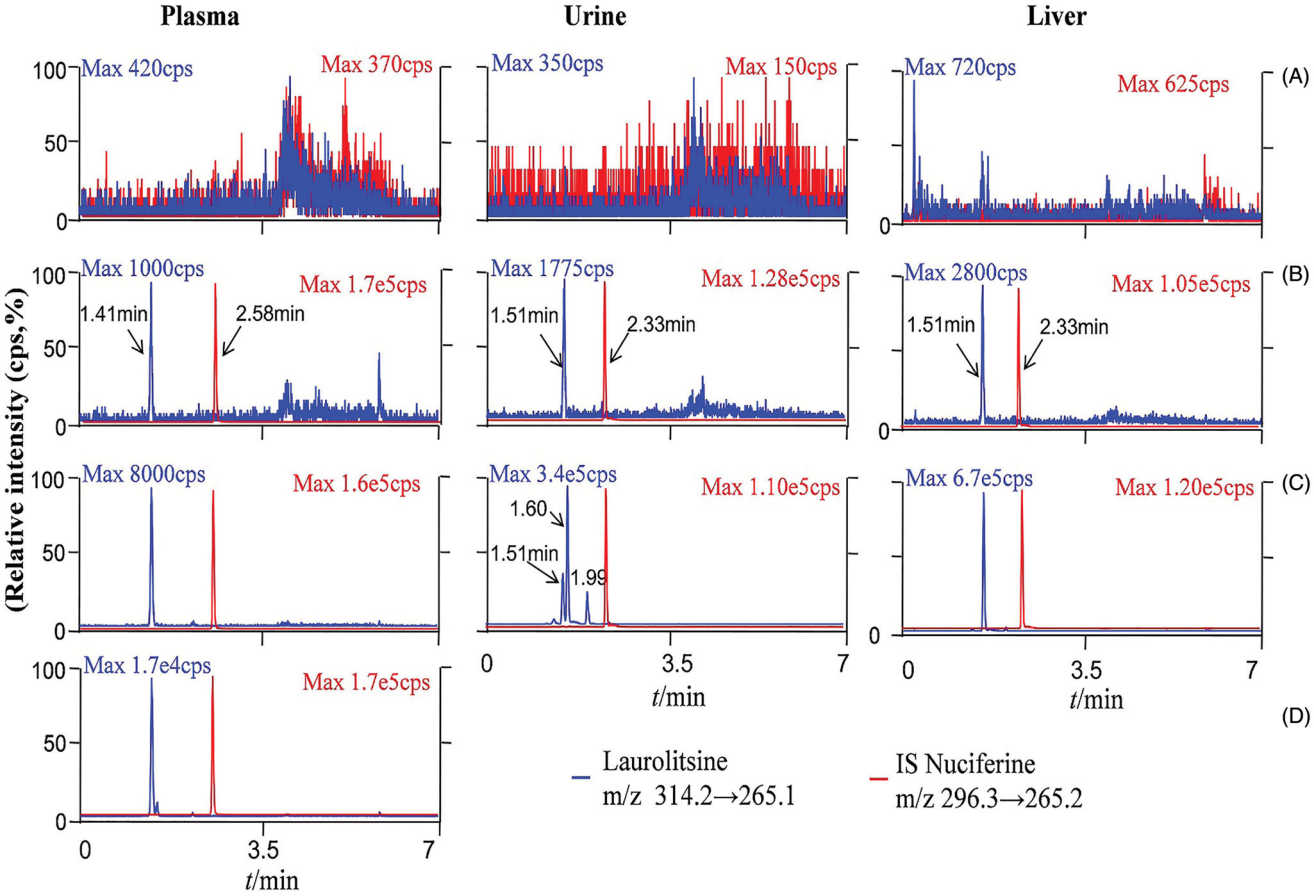
LC-MS/MS chromatograms oflaurolitsine and IS nuciferine in different matrix. (A) Blank biological matrix; (B) LLOQ sample of different biological matrix (laurolistine 1 ng/mL^−1^); (C) rat plasma of 1 h after i.v. administration laurolistine, rat urine of 0–4 h after p.o. administration laurolistine, rat liver tissue of 2 h after p.o. administration laurolistine; (D) rat plasma of 1 h after p.o. administration laurolitsine.

Representative chromatograms of blank biological matrix, blank biological matrix spiked with laurolitsine and IS, and rat samples are shown in Figure 2. Laurolitsine and IS were separated well without cross interference. The retention times of laurolitsine and IS were 1.41 min and 2.58 min, respectively, for plasma and 1.51 and 2.33 min for urine, faeces and tissue samples. The carryover effect also did not appear in this method. The residual laurolitsine peak area in blank biomatrix samples was less than 20% of the peak area of the LLOQ sample, and the residual IS peak area was less than 5% of the QC sample.

#### Linearity

The standard calibration curve for laurolitsine in all biological samples has a good linear relationship. The regression equation, correlation coefficient and quantitative range are shown in Table 1.

**Table 1.** Standard calibration curves and LLOQ of laurolitsine in different matrix.

Matrix	Equation (1/x^2^, Weighting Index)	Range (ng/mL)	LLOQ (ng/mL)
Plasma	y = 0.00741x + 0.00319 (*r* = 0.9979)	1–2000	1
Liver	y = 0.01438x − 0.00754 (*r* = 0.99885)	1–2000	1
Kidney	y = 0.01238x − 0.00283 (*r* = 0.99935)	1–2000	1
Spleen	y = 0.01566x + 0.00106 (*r* = 0.99968)	1–2000	1
Lung	y = 0.01359x − 0.00366 (*r* = 0.99938)	1–2000	1
Brain	y = 0.01704x − 0.00697 (*r* = 0.99804)	1–2000	1
Heart	y = 0.01385x + 0.00144 (*r* = 0.99583)	1–2000	1
Testis	y = 0.01286x − 0.00393 (*r* = 0.99929)	1–2000	1
Stomach	y = 0.01060x + 0.00292 (*r* = 0.99812)	5–10000	5
Muscle	y = 0.01717x − 0.00045 (*r* = 0.99838)	1–2000	1
Fat	y = 0.01771x − 0.00126 (*r* = 0.99944)	1–2000	1
Intestine	y = 0.01258x − 0.00023 (*r* = 0.99687)	1–2000	1
Urine	y = 0.0122x − 0.01301 (*r* = 0.9984)	1–2000	1
Faeces	y = 0.0153x + 0.0203 (*r* = 0.9994)	5–10000	5

#### Precision, accuracy and stability

The intraday and interday precision were measured by relative standard deviation (RSD), and the values were both lower than 15%, as shown in Table 2. The accuracy of the laurolitsine test during our results ranged from 90.95 to 110.2%, as shown in Table 2. All our data suggested that the detection method of laurolitsine has high precision and accuracy in this study (Table 2). We further tested the stability of laurolitsine in plasma, liver, kidney, urine, and faeces under different conditions, such as storage temperature, storage time and freeze–thaw treatment. The precision was also measured by the relative standard deviation (RSD). As shown in Table 3, the RSD values were within ±11.45% for the stability of laurolitsine at the three levels of QC plasma samples. For tissue samples, the liver and kidney were listed as representatives.

**Table 2.** Intra-day and inter-day precision, accuracy of laurolitsine.

Matrix	Laurolitsine Spiked (ng/mL^−1^)	Intra-day (*n* = 6)		Accuracy (%)	Inter-day (*n* = 18)		Accuracy (%)
		Measured (ng/mL)	RSD (%)		Measured (ng/mL)	RSD (%)	
Plasma	3	3.30 ± 0.21	6.25	110.2	3.18 ± 0.24	7.45	105.88
	120	118.24 ± 5.51	4.66	98.54	118.49 ± 6.55	5.53	98.74
	1500	1513.38 ± 97.21	6.42	100.89	1495.96 ± 90.69	6.06	99.73
Liver	3	2.92 ± 0.25	8.62	97.30	2.97 ± 0.23	7.61	99.07
	150	161.03 ± 6.55	4.07	107.36	156.64 ± 8.42	5.38	104.43
	1500	1445.28 ± 84.33	5.83	96.35	1505.16 ± 111.74	7.42	100.34
Kidney	3	2.93 ± 0.28	9.72	97.65	3.02 ± 0.29	9.57	100.59
	150	153.88 ± 9.91	6.44	102.59	154.79 ± 8.86	5.73	103.20
	1500	1429.94 ± 72.77	5.09	95.33	1485.20 ± 96.66	6.51	99.01
Urine	3	3.21 ± 0.22	6.93	106.83	3.18 ± 0.23	7.10	105.98
	150	149.95 ± 12.17	8.11	99.97	151.80 ± 8.97	5.91	101.20
	1500	1508.78 ± 98.85	6.55	100.59	1527.17 ± 84.28	5.52	101.81
Faeces	12	11.78 ± 0.97	8.26	96.10	11.89 ± 0.94	7.92	99.11
	750	723.19 ± 54.12	7.48	90.95	728.39 ± 51.10	7.02	97.12
	7500	7465.22 ± 315.39	4.22	98.13	7470.99 ± 301.06	4.03	99.61

**Table 3.** Stability of laurolitsine in plasma, liver, kidney, urine, faeces (*n* = 6).

Matrix	Investigation conditions	Theoretical concentration (ng/mL)	Measured concentration (Mean ± SD) (ng/mL)	RSD (%)	Accuracy (%)
Plasma	Room temperature stability (25 °C, 6 h)	3.00	2.99 ± 0.22	7.47	99.67
		120.00	117.90 ± 6.87	5.83	98.25
		1500.00	1591.77 ± 90.47	5.68	106.12
	Short time stability (4 °C, 24 h)	3.00	3.04 ± 0.14	4.48	101.33
		120.00	119.31 ± 5.60	4.70	99.43
		1500.00	1542.88 ± 56.57	3.67	102.86
	Freeze thaw stability (12 h/12 h)	3.00	3.10 ± 0.24	7.63	103.33
		120.00	126.27 ± 5.32	4.21	105.23
		1500.00	1404.27 ± 62.12	4.42	93.62
	Long time freezing stability (−20 °C, 7 d)	3.00	3.07 ± 0.26	8.35	102.33
		120.00	121.55 ± 8.67	7.12	101.29
		1500.00	1456.63 ± 61.21	4.20	97.11
Liver	Room temperature stability (25 °C, 6 h)	3.00	3.06 ± 0.14	4.65	102.00
		150.00	150.43 ± 9.86	6.55	100.29
		1500.00	1526.32 ± 86.93	5.70	101.75
	Short time stability (4 °C, 24 h)	3.00	2.97 ± 0.29	9.65	99.00
		150.00	152.75 ± 10.95	7.17	101.83
		1500.00	1519.98 ± 110.13	7.25	101.33
	Freeze thaw stability (12h/12 h)	3.00	3.15 ± 0.24	7.69	105.00
		150.00	149.51 ± 15.07	10.08	99.67
		1500.00	1519.96 ± 151.86	9.99	101.33
	Long time freezing stability (−20 °C, 7 d)	3.00	3.03 ± 0.28	9.34	101.00
		150.00	147.36 ± 15.47	10.50	98.24
		1500.00	1554.67 ± 122.62	7.89	103.64
Kidney	Room temperature stability (25 °C, 6 h)	3.00	3.05 ± 0.13	4.39	101.67
		150.00	149.70 ± 7.28	4.87	99.80
		1500.00	1515.43 ± 5.84	5.84	101.03
	Short time stability (4 °C, 24 h)	3.00	2.97 ± 0.26	8.90	99.00
		150.00	147.70 ± 11.28	7.63	98.47
		1500.00	1476.49 ± 78.73	5.33	98.43
	Freeze thaw stability (12h/12 h)	3.00	2.97 ± 0.27	8.91	99.00
		150.00	151.00 ± 11.41	7.56	100.67
		1500.00	1475.38 ± 142.92	9.69	98.36
	Long time freezing stability (−20 °C, 7 d)	3.00	3.06 ± 0.28	7.65	102.00
		150.00	145.12 ± 16.62	11.45	96.75
		1500.00	1493.25 ± 145.80	9.76	99.55
Urine	Room temperature stability (25 °C, 6 h)	3.00	3.03 ± 0.10	3.35	101.00
		150.00	148.71 ± 6.97	4.69	99.14
		1500.00	1509.65 ± 77.34	5.12	100.64
	Short time stability (4 °C, 24 h)	3.00	3.02 ± 0.28	9.34	100.67
		150.00	149.58 ± 7.27	4.86	99.72
		1500.00	1472.03 ± 111.54	7.58	98.14
	Freeze thaw stability (12 h/12 h)	3.00	3.02 ± 0.32	10.62	100.67
		150.00	151.50 ± 13.45	8.88	101.00
		1500.00	1477.47 ± 114.99	7.78	98.50
	Long time freezing stability (−20 °C, 7 d)	3.00	3.08 ± 0.20	6.50	102.67
		150.00	148.02 ± 13.57	9.17	98.68
		1500.00	1522.16 ± 91.75	6.03	101.48
Faeces	Room temperature stability (25 °C, 6 h)	12.00	3.05 ± 0.13	6.14	25.42
		750.00	149.70 ± 7.28	5.24	19.96
		7500.00	1515.43 ± 5.84	3.36	20.21
	Short time stability (4 °C, 24 h)	12.00	2.97 ± 0.26	11.37	24.75
		750.00	147.70 ± 11.28	4.21	19.69
		7500.00	1476.49 ± 78.73	4.22	19.69
	Freeze thaw stability (12 h/12 h)	12.00	2.97 ± 0.27	9.18	24.75
		750.00	151.00 ± 11.41	6.23	20.13
		7500.00	1475.38 ± 142.92	3.76	19.67
	Long time freezing stability (−20 °C, 7 d)	12.00	3.06 ± 0.28	8.24	25.50
		750.00	145.12 ± 16.62	8.22	19.35
		7500.00	1493.25 ± 145.80	4.65	19.91

#### Extraction recovery and matrix effect

The extraction recovery and matrix effects of laurolitsine were within acceptable criteria for the assay of the analyte in biological samples from rats. There were no significant differences in matrix effects among the different biological samples from rats. The results of extraction recovery and matrix effect are listed in Table 4.

**Table 4.** Extraction recovery and matrix effect of laurolitsine (Mean ± SD, *n* = 6).

Matrix	Concentration (ng/mL)	Extraction recovery (%)	Matrix effect (%)
Plasma	3.00	89.00 ± 4.52	91.99 ± 11.10
	120.00	92.51 ± 6.52	93.67 ± 2.63
	1500.00	92.84 ± 10.41	95.94 ± 9.23
Liver	3.00	89.74 ± 4.01	97.87 ± 9.33
	150.00	94.39 ± 8.33	96.33 ± 4.81
	1500.00	91.22 ± 6.87	96.87 ± 7.95
Kidney	3.00	87.37 ± 5.78	101.14 ± 6.83
	150.00	90.24 ± 6.75	93.01 ± 5.90
	1500.00	95.21 ± 5.85	96.92 ± 7.80
Urine	3.00	91.68 ± 5.22	95.82 ± 8.09
	150.00	94.13 ± 6.34	96.92 ± 4.86
	1500.00	93.05 ± 6.53	101.69 ± 4.09
Faeces	12.00	93.25 ± 7.97	103.21 ± 9.71
	750.00	87.13 ± 8.32	98.97 ± 6.51
	7500.00	93.05 ± 5.75	97.54 ± 7.16

### Pharmacokinetics of laurolitsine in rats

**Figure 3.**
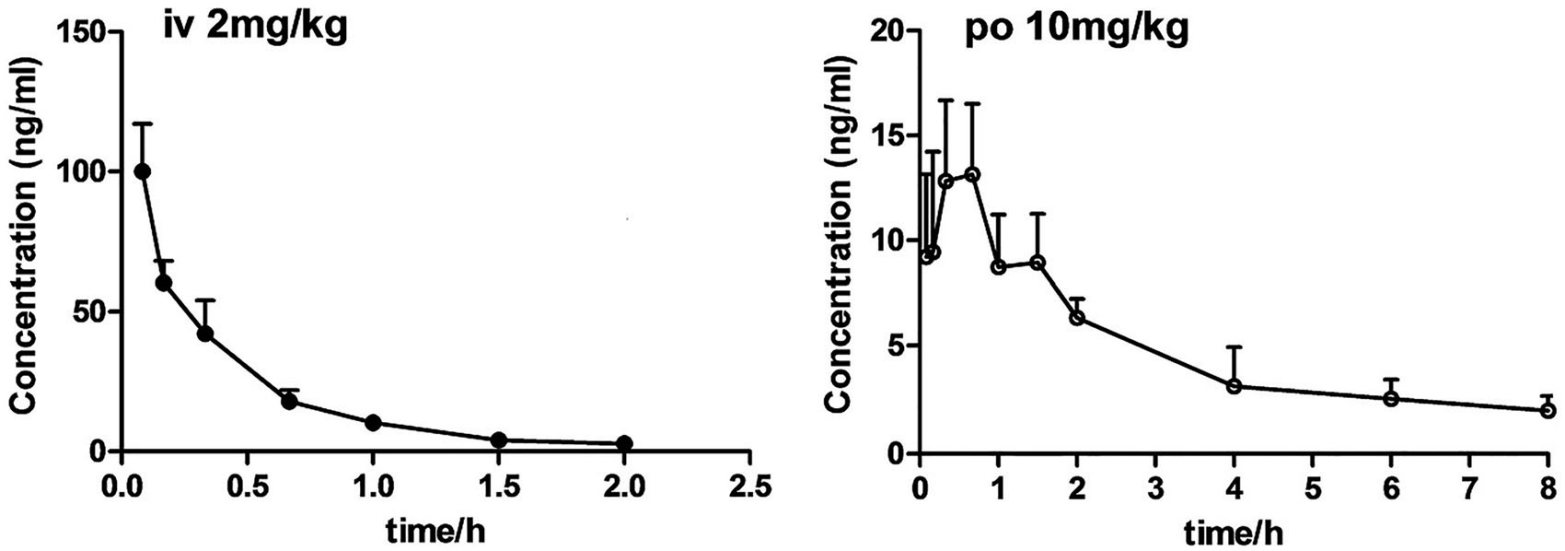
Plasma concentration-time curve of laurolitsine after i.g. (10 mg/kg) and i.v. (2 mg/kg) administration to SD rats (*n* = 5).

The developed UPLC-MS/MS method was applied to the pharmacokinetic analysis of laurolitsine. The mean plasma concentration-time curves for laurolitsine after a single dose administration (*n* = 5) are shown in Figure 3, and the main noncompartment pharmacokinetic parameters are summarised in Table 5. The maximum concentration (*C*_max_) of laurolitsine was 100.05 ng/mL after *i.v.* administration at 0.083 h. Then, it decreased rapidly to very low, and after 4 h, it fell below the LLOQ (1 ng/mL) (Figure 3). On the other hand, the *C*_max_ was 14.11 ng/mg after *i.g*. administration at 0.47 h, indicating that the absorption of laurolitsine was faster (Figure 3). The bioavailability of oral administration was 18.17% and low in rats.

**Table 5.** Pharmacokinetic parameters of laurolitsine after *i.g.* and *i.v.* admininstration.

PK parameters	*i.v.* Admininstration (2 mg/kg)	*i.g*. Admininstration (10 mg/kg)
t_1/2_ (h)	1.67 ± 1.83	3.73 ± 2.48
C_max_ (ng/mL)	100.05 ± 17.00	14.11 ± 3.30
T_max_(h)	0.083	0.47 ± 0.18
AUC_0-t_ (h·ng/mL)	49.98 ± 7.39	40.62 ± 9.57
AUC_0-∞_ (h·ng/mL)	52.29 ± 7.98	47.77 ± 10.44
MRT _(0-t)_(h)	0.61 ± 0.22	2.90 ± 0.53
Vz(L/kg)	86.04 ± 86.75	1207.59 ± 799.48
CLz (L/h/kg)	38.96 ± 5.86	218.26 ± 51.89
F(%)	–	18.17

### Tissue distribution of laurolitsine

**Figure 4.**
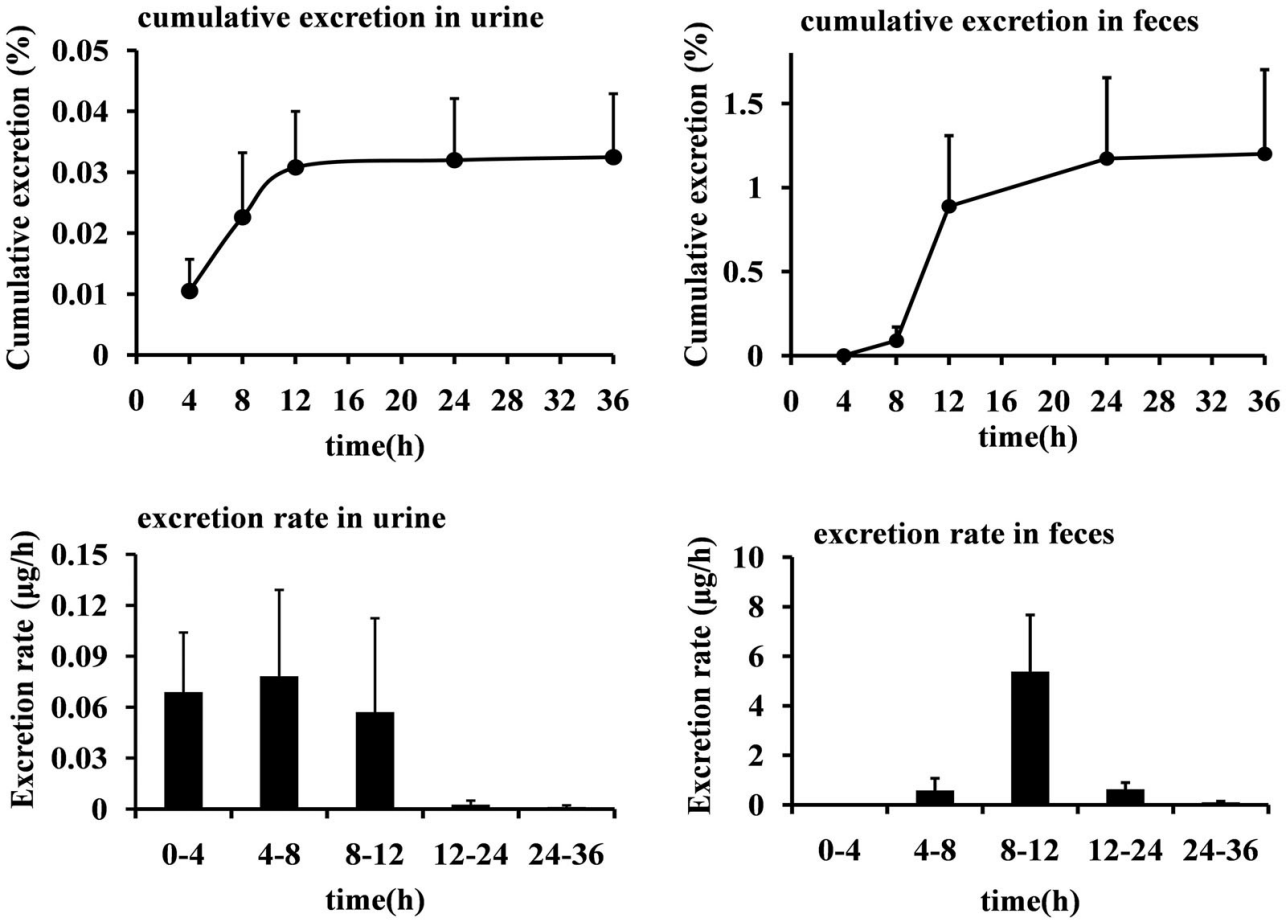
The mean tissue drug concentrations at 0.5, 1, 2, 4, and 6 h after the oral administration of 10 mg/kg laurolitsine.

The tissue distribution of laurolitsine in rats at 0.5, 1, 2, 4 and 6 h after the oral administration of 10 mg/kg is presented in Figure 4. Laurolitsine can be rapidly and widely distributed in various tissues of the rat as a prototype. Laurolitsine is highly concentrated in the stomach and intestine and is widely detected in the liver, lungs and kidneys, suggesting that most laurolitsine is quickly absorbed into the bloodstream by the stomach and intestine orally and quickly distributed in various tissues, such as the liver, lungs and kidneys. Laurolitsine was also detected in the brain and indicated that it can pass through the blood-brain barrier, but the concentration was low. At the same time, the experimental results found that laurolitsine reached its peak in all tissues at 30 min, after which the concentration slowly decreased, while the drug concentration of laurolitsine in the liver and small intestine rose slightly at 2 h. 6 h after administration, laurolitsine could not be detected in brain, muscle, and testis tissues, and the drug concentration in all tissues except the liver fell below 10% of the peak concentration, indicating that laurolitsine does not easily accumulate in the body.

### Excretion of laurolitsine in rats

**Figure 5.**
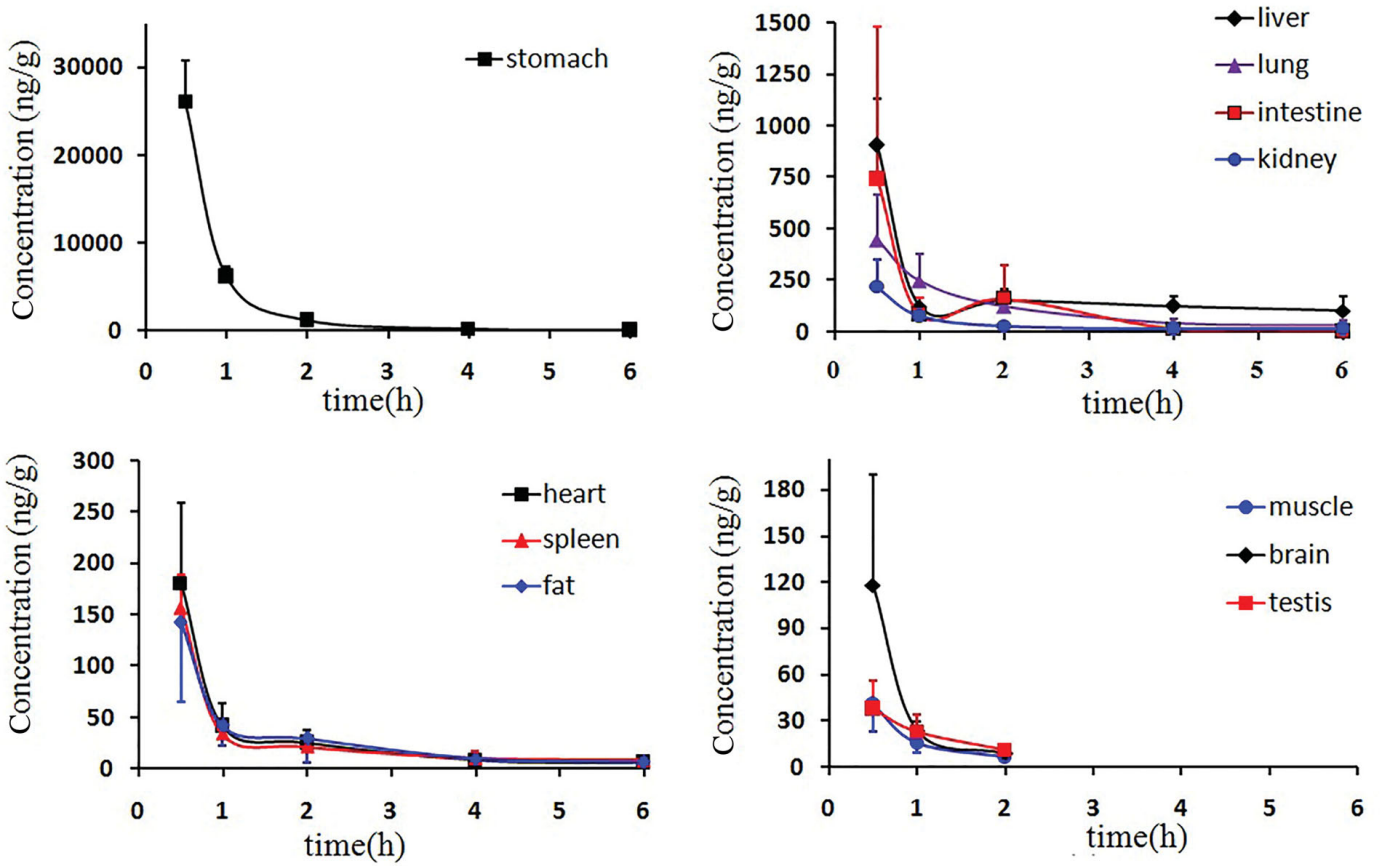
The cumulative excretion of laurolitsine in the urine and faeces of rats after oral administration 10 mg/kg as a percentage of the dosage and excretion rate.

The urinary and faecal cumulative excretion rates of laurolitsine in rats after oral administration of 10 mg/kg are shown in Figure 5. After rats were given 10 mg/kg laurolitsine by gavage, their urine reached the maximum excretion rate at 4–8 h and faeces at 8–12 h. Within 36 h, the prototype laurolitsine excreted 0.0325% of the dose through urine and 1.20% through faeces. Excretion is very low by both routes.

## Discussion

A stable, high-throughput, accurate and sensitive determination method is necessary and important for the study of pharmacokinetics and tissue distribution. In this study, the UPLC-MS/MS quantitative analysis method based on triple quadrupole mass spectrometry was developed based on previous research (Fortuna et al. 2013). We first tested the MRM signal response intensity of laurolitsine and the IS nuciferine in ESI positive ion and negative ion modes to choose the optimal mode; we ultimately selected the positive ion mode by optimizing a series of parameters, such as DP, CE, GS1, GS2, IS, TEM, etc. Then, we compared the elution effects of using methanol-water and acetonitrile-water systems as the mobile phase and compared the addition of different concentrations of formic acid to the mobile phase. Adjust the elution gradient program of the mobile phases. Finally, acetonitrile-water containing 0.5‰ formic acid was selected as the best mobile phase. For plasma samples, a gradient elution procedure under the item “Sample preparation” was used; for urine, faeces, and tissue samples, precolumns were used, and the gradient elution procedure was fine-tuned because there were many impurities. The gradient elution procedure was fine-tuned as follows: solvent A (water containing 0.5‰ formic acid) and solvent B (acetonitrile containing 0.5‰ formic acid), 5% B (0 min), 5–45% B (0–3 min), 45–95% B (3–3.5 min), 95% B (3.5–5 min), and 5% B (5.1–6.5 min). Comparing the pre-treatment of the samples with methanol and acetonitrile to precipitate the protein, it is found that the chromatogram peak shape of the sample treated with methanol is better, the interference is less, and the signal response is strong. Therefore, methanol was used for subsequent experiments.

By revealing the absorption and distribution features at different time points, we can provide a good understanding and reference for the pharmacological efficacy of laurolitsine and its potential role in target research during our further work.

The above results indicate that laurolitsine may have a first-pass effect in the liver after oral administration; on the other hand, the prototype drug undergoes extensive metabolic transformation, resulting in a small amount of laurolitsine that enters the body circulation after oral administration, and the blood concentration in the body is very low. The excretion from urine and faeces will also be reduced. As shown in Figure 2, in the LC-MS/MS chromatogram of rat urine, the parent drug could be detected with a retention time (Rt) of approximately 1.51 min, and there were two peaks at 1.60 and 1.99 min. These peaks appear to reflect metabolites of laurolitsine.

In pharmacokinetic studies of other aporphine alkaloids, glucuronidation and sulphation may be the two main metabolic pathways. For example, the metabolites of norisoboldine in urine and bile include noriosboldine-1-*O-β*-D-glucuronide, norisoboldine-9-*O-α-*D-glucuronide and disulphuric acid-1,9-norisoboldine ester (Chen et al. 2010). The metabolites of isoboldine include monosulfate-isoboldine, disulfate-isoboldine, isoboldine-monoglucuronide, and monosulfate-isoboldine-monoglucuronide (Li et al. 2015). Laurolitsine has a similar structure to isoboldine and is an aporphine alkaloid (Torres-Vega et al. 2020). Therefore, we believe that laurolitsine may also metabolize through the glucuronidation or sulphation pathways, but this possibility needs to be explored further in our future work.

## Conclusions

In the present assay, a simple, rapid and accurate LC-MS/MS method was established and validated for the qualification of laurolitsine in rat plasma, tissues, urine and faeces. These methods were successfully applied to pharmacokinetics, tissue distribution and excretion. Our work is the first to report the pharmacokinetic characteristics of laurolitsine in rats. Laurolitsine is easily absorbed by the stomach and intestine; distributed quickly to the liver, lungs, and kidneys; and then completely metabolised after 4 h with relatively low oral bioavailability. Only a small amount of laurolitsine is mainly excreted through faeces. From the above results, we have a good understanding of the pharmacokinetics, tissue distribution and excretion of laurolitsine and found that those parameters are not perfect. Therefore, the structural modification of laurolitsine is necessary to improve its pharmacokinetic properties, which we will also address in our further work.
